# Jointing Achievement and Performance Evaluation of Bogie Crossmember Ring Joint Welded via Inertia Friction Welding

**DOI:** 10.3390/ma16227127

**Published:** 2023-11-10

**Authors:** Feng Qin, Xinmeng Zhang, Chunbo Zhang, Yanquan Wu, Wu Liang, Rui Li, Jun Zhou

**Affiliations:** 1 Harbin Welding Institute Limited Company, Harbin 150028, China; 2 Heilongjiang Advanced Friction Welding Technology and Equipment Key Laboratory, Harbin 150028, China; 3 CRRC Changchun Railway Vehicles Co., Ltd., Changchun 130062, China

**Keywords:** inertia friction welding, microstructure, mechanical properties

## Abstract

As a major load-bearing component of trains, the weld quality of the bogie beam is critical to the safety of railway operations. This study specifically investigates the inertia friction welding process of S355 bogie crosshead tubes, with the aim of improving the weld quality and achieving one-time formation of the crosshead tube and tube seat. The microstructural features and mechanical properties of S355 inertia-welded joints were also compared with the base metal. Research indicates that inertia friction welds have no visible defects, and that the microstructure of the welding seam (WS) consists of granular bainite, acicular ferrite and little pearlite. The thermo-mechanically affected zone (TMAZ) consists of granular bainite bands and ferrite + pearlite bands. The hot work strengthening mechanism of inertia friction welding results in a higher level of hardness for both WS and TMAZ. The tensile property of the welded joints can be compared to the base metal. The yield strength, tensile strength and elongation of the welded joints, respectively, reach 87.5%, 100% and 79.5% of S355. However, the impact toughness of the welds at room temperature is lower than that of the base material, particularly in the TMAZ zone. Conversely, in an environment with a temperature of −40 °C, WS’s impact toughness surpasses that of the parent material.

## 1. Introduction

Train bogies composed of various forms of welding structures significantly affect trains’ safety performance and serviceability. The crossmember part of S355 steel, which consist of a tube and two tube holders at each end, affiliate with and pass through the entire bogie. Between the tube and two holders, there exist two circular welded joints, and since the crossmember part connects the two side beams and carries the traction, brake and vibration damping devices, these two ring welding seams are classified as critical joints for the bogie. Typically, double-sided grooves are applied to these ring joins and welded via multi-layer and multi-pass arc welding. However, these welding seams often demonstrate a low NDT pass rate because of their internal defects, such as porosity, incomplete fusion and cinder inclusion, which seriously reduce the welding quality. Furthermore, the ensuing extensive repair efforts seriously delay the overall production pace and hinder the whole bogie manufacturing process.

With regard to bogie welding, a number of researchers have used numerical analysis to predict and evaluate residual stress, welding deformation and fatigue performance. Ilesanmi et al. [1] used Abaqus software 2018 to model and simulate a railcar bogie and used Taguchi’s method to analyze the impact of welding parameters on the welding deformation model. The results showed that the input welding parameters are key factors and have a significant impact on the welding process, and therefore need to be controlled. Lu et al. [2] established two kinds of finite element models to analyze the weld deformation of single-sided, two-pass-welded T-joints. The results indicated that the numerical simulation values using these two methods are generally in accordance. In addition, the welding fatigue performance of the bogie is also a concern for researchers. Shukri et al. [3] established a new fatigue model based on the endurance limit approach and using residual stresses to predict the S-N curves. Gao et al. [4] constructed welded railway vehicle structures on the basis of the kriging surrogate model and conducted lightweight optimization subject to weld fatigue and static strength constraints. Seo et al. [5] compared the fatigue performance of railway vehicle bogies with SM490A material before and after repair welding and conducted finite element analysis to evaluate the residual stress change. Zhao et al. [6] carried out fatigue property tests of typical welded joints and studied the effects of joint dimensions to obtain a safer S-N curve. Yang et al. [7] developed a new method to calculate equivalent stress amplitudes to assess fatigue damage based on measurable randomness.

Researchers have also conducted systematic studies on the welding of S355 steel. On S355 steel weld fatigue crack extension, Mao et al. [8] investigated the S355 steel weldment behavior of fatigue cracks in the welded details of motor hangers through crack growth numerical simulation under constant amplitude loading. Dariusz et al. [9] researched the rate of fatigue crack growth in fillet welds of S355 steel subjected to bending. Regarding post-weld residual stress improvement, for post-weld residual stress improvement, high-frequency vibrations [10,11,12] were used in S355 steel weldment, and the results showed that high-frequency mechanical impact significantly extended the fatigue life. In terms of welding process, Wu et al. [13] used compound external magnetic field-assisted GMAW to manufacture S355J2W steel T-joint bogie structures, and the result showed that this assisted method can suppress spatter formation and undercut defects. In several studies [14,15,16], the technique of friction taper plug welding was employed to weld S355 steel, and the microstructural and mechanical characteristics of the welded joints were investigated.

Overall, most of the above studies have been based on arc welding, and fewer of them have aimed to address the challenge of avoiding bogie welding defects. As a solution to the above problem in this research, inertia friction welding as a solid-phase welding method was utilized to achieve the one-time formation of a bogie crossmember ring joint. Due to its solid-phase joining process, the welded structure barely contains melting defects such as porosity and slag. In this study, the welded joints were evaluated for their tensile properties and ductility, and their impact properties and microhardness in comparison with the base metal, while microstructural analyses were carried out on each of the microscopic areas of the welded joints.

## 2. Materials and Methods

S355 pipes and forgings using normalized fine-grained structural steel with good impact toughness and welding performance were welded in this study. Chemical composition and mechanical properties are given in Table 1.

**Figure 1:**
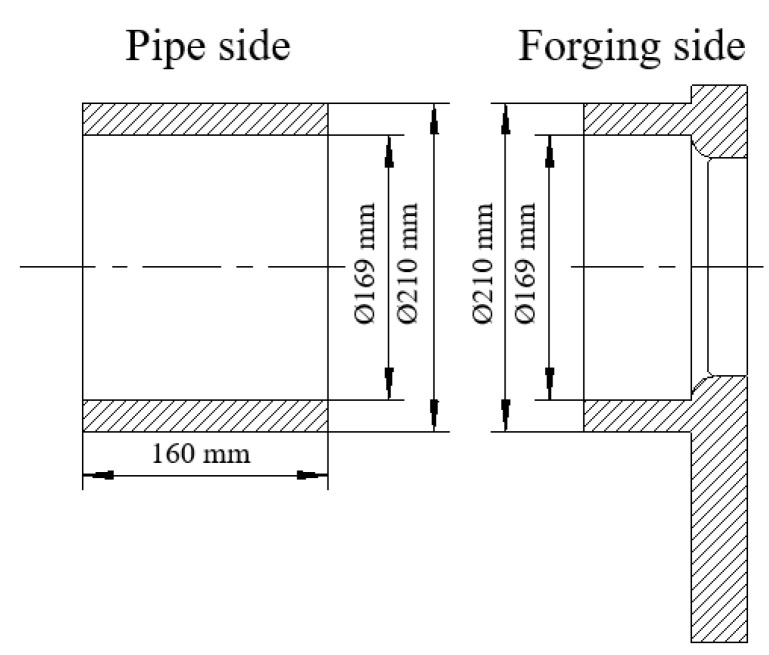
Size of workpiece joints.

**Figure 2:**
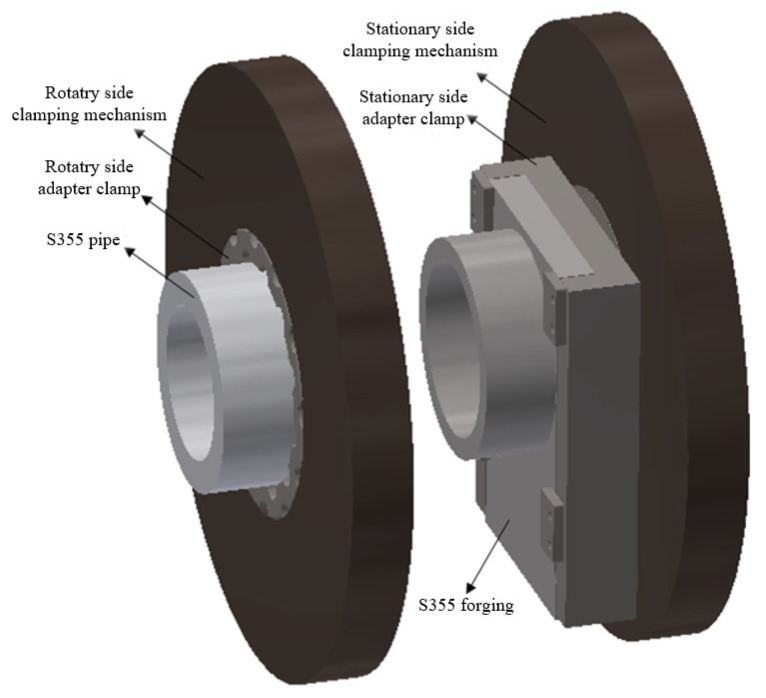
Schematic diagram of workpiece clamping type.

The geometry of the welded workpiece is shown in Figure 1. The thickness of the welded joint is 20.5 mm. Due to the characteristic of non-center symmetry of the forging, this part was fixed at the non-rotating end using special jigs and fixtures, parallel with the steel pipe fittings mounted on the rotating end. Figure 2 details the workpiece clamp status.

**Figure 3:**
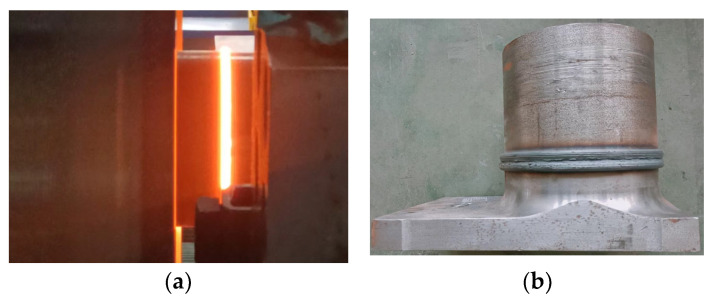
Inertia friction welding process and welded joint after welding: (**a**) red hot joints during the inertia friction welding process; (**b**) general appearance of the joint after welding.

The whole welding process was carried out using an HWI-GHX-600 high-precision inertia friction welding machine manufactured by the Harbin Welding Research Institute (Harbin, China). Before welding, the two side welding surfaces were polished to remove rust and the moment of inertia was adjusted to 3500 kg·m^2^. At the same time, the spindle speed and upsetting pressure were set to 330 rpm and 150 MPa, respectively. The inertia friction welding process and the visual appearance of the welded joints are shown in Figure 3.

**Figure 4:**
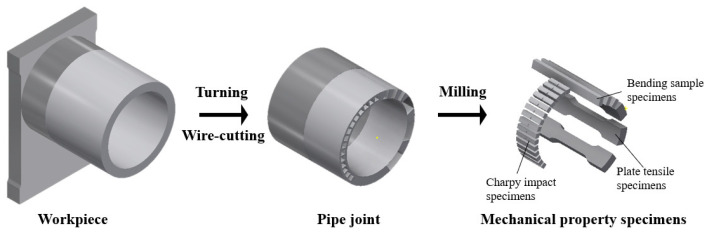
Schematic of test piece processing steps.

After welding, the workpiece was subjected to a stress-alleviation heat treatment at 530 °C for a duration of 3 h. Subsequently turning, wire-cutting and milling processes were employed to create specimens for performance testing; the specific machining process of the specimens is shown in Figure 4. Full-thickness plate tensile testing and joint bending testing were carried out three times under the requirements of relevant standard [17,18] using WE-600B universal testing machine (Jinan, China). Furthermore, the weld seam (WS), thermo-mechanically affected zone (TMAZ) and base metal (BM) were each subjected to a series of impact tests at both room temperature and low temperature (−40 °C) in accordance with standard [19] using ZBC2452-C pendulum impact testing machine (Shenzhen, China). Each group comprised three specimens, and the test results were averaged from the three specimens. Vickers microhardness testing was carried out on the bonding interface at a force of 9.861 N for 10 s using HVS-1000 microhardness tester (Dezhou, China). The Axiovert 40 MAT optical microscope and Zeiss supra 55 scanning electron microscopy (Jena, Germany) were used to observe the microstructure of different regions of the welded joint, the WS and part of the TMAZ were scanned via energy spectrum scanning, and metallographic samples were etched with a 4% nitric acid solution. It should be noted that an equal number of full-thickness plate tensile testing samples were taken from the base material to serve as a comparison group.

## 3. Results

### 3.1. Macroscopic and Microstructural

#### 3.1.1. Flash Characteristics

In the rotary friction welding process, flashing is formed through the combined action of heat and pressure resulting from the friction between the welded materials, manifesting as extrusion of material at the edge of the weld. The appearance of flash can help to determine the quality of the weld.

**Figure 5:**
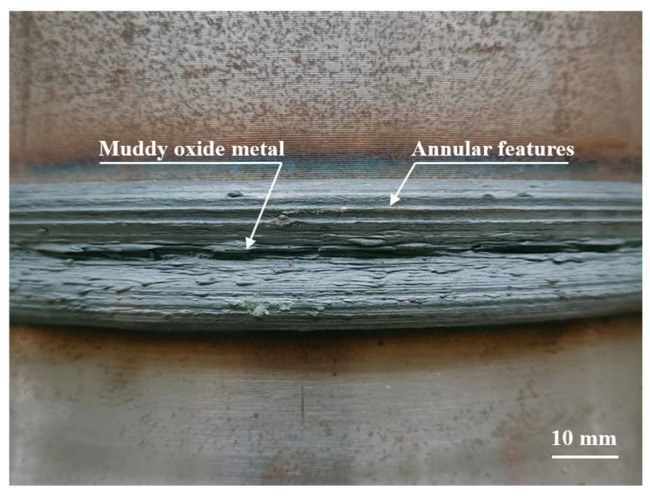
Surface morphology of flash.

The morphology of the welded joint flash is illustrated in Figure 5. Flash surfaces have a rough texture and oxidized color characteristics. The presence of surface annular features and muddy oxide metal suggests sufficient forging pressure and friction heat during welding.

#### 3.1.2. Macro Metallography

**Figure 6:**
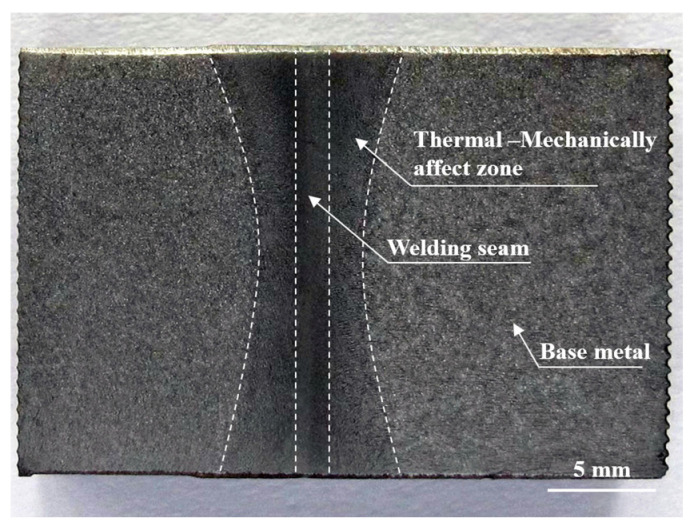
Macroscopic metallography of inertia friction-welded joint.

Figure 6 displays the macroscopic metallography of the S355 inertia friction-welded joint. Due to the friction pressure on the thermoplastic metal, flash forms on the inner and outer surfaces of the weld, resulting in an hourglass-shaped weld feature. Furthermore, the weld exhibits no defects, including those classified as incomplete bonding, inclusions and cracks.

In the workpiece axis direction, the frictional heat and forging forces also differ, producing welds with different subarea characteristics. The central part is the welding seam zone, which has the highest friction temperature during the welding process, closest to the welding interface, leading to a vertically elongated zone. Adjacent to the welding seam is the thermo-mechanically affect zone, which is formed when the thermoplastic metal is compressed by the forging forces. The base metal area of the joint is on the outside of the weld area.

#### 3.1.3. Microstructure

**Figure 7:**
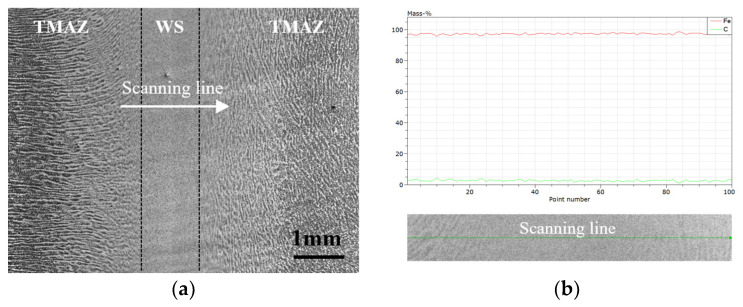
Low-power SEM image and line scan results near weld center: (**a**) low-power secondary electron imaging of weld center; (**b**) energy spectrum scanning results across WS.

The microstructure morphology is different to that of fusion welding [20,21,22,23], and also to that of forging, due to the fact that inertia friction welding is a solid joining process and the base material does not melt during welding. Figure 7 illustrates the low-power microstructure of the weld center under the scanning electron microscope. The microstructure of the WS is uniform and the TMAZ shows obvious flow line characteristics, whereby the direction of the flow line extension matches the direction of the thermoplastic metal flow direction during upsetting. According to the analysis of the energy spectrum scanning results across the WS region, the uneven microstructure transition from WS to TMAZ did not cause segregation of the C element.

**Figure 8:**
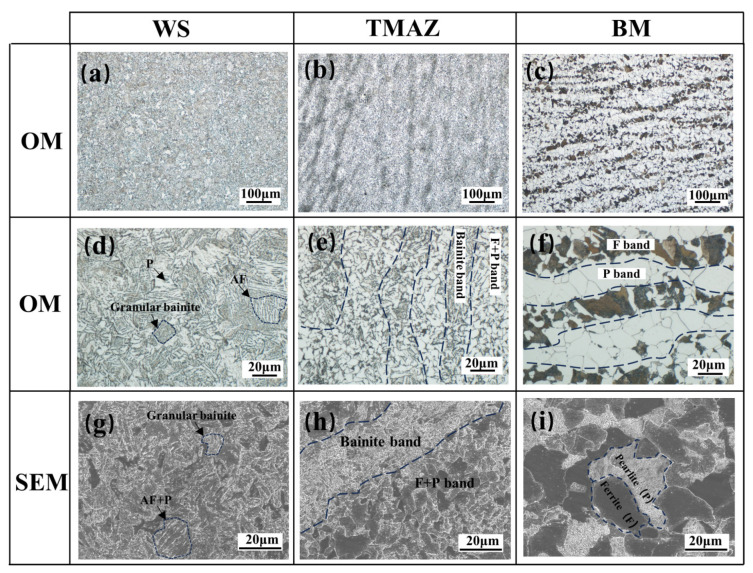
Microstructure characteristics of different regions of S355 inertia friction-welded joints: (**a**) WS under 100× OM; (**b**) TMAZ under 100× OM; (**c**) BM under 100× OM; (**d**) WS under 500× OM; (**e**) TMAZ under 500× OM; (**f**) BM under 500× OM; (**g**) WS under 1000× SEM; (**h**) TMAZ under 1000× SEM; (**i**) BM under 1000× SEM.

Figure 8 shows the optical microscope (OM) and scanning electron microscope (SEM) images of microstructural and morphological characteristics of different regions of the welded joint. As the S355 workpiece was supplied as a forging, the microstructure of the parent material is ferrite and pearlite overlapped in a band-like microstructure feature in Figure 8c,f, and the black ferrite (F) grains and gray pearlite (P) grains can be clearly seen in Figure 8i.

As the IFW process remains below the melting point of the base material and most base material forgings act as a heat absorption sink, the cooling rate is faster than the normalizing heat-treatment process. Consequently, TMAZ does not generate the typical F+P normalized homogeneous fine-grained microstructure during air cooling after exposure to a high temperature between Ac_1_ and Ac_3_ [24]. Instead, a kind of F+P equiaxed fine-grained band interlayered with a bainite band forms in the TMAZ zone, as shown in Figure 8b,h. Since the plastically deformed metal of TMAZ forms a carbide segregation zone during hot working under the IFW mechanism, these zones experience a complete austenitizing process, and during the following rapid cooling process, the austenite band zone evolves into supercooled ferrite, eventually forming a granular bainite band. At the same time, the carbide-poor band experiences a recrystallisation process [25] and forms an F+P equiaxial fine-grained microstructure. The proportion of P in the F+P band is significantly reduced compared to the base metal, as seen in Figure 8e.

The WS region experiences the fullest plastic deformation and the highest frictional heat cycle temperature, exceeding Ac_3_ [24], so that the phase transition drive is at the highest level without significant carbon segregation. This leads to a homogeneous mixed microstructure of granular bainite with acicular ferrite (AF) and a small amount of pearlite, as shown in Figure 8a,d. Since WS does not have obvious large-angle grain boundary features and the boundaries between grains are difficult to distinguish in Figure 8g, it is presumed that there are a high number of substructures in this region, and as a reinforcement mechanism for the joints, this inference will be verified later in the microhardness analysis section.

### 3.2. Mechanical Properties

#### 3.2.1. Microhardness Distribution

**Figure 9:**
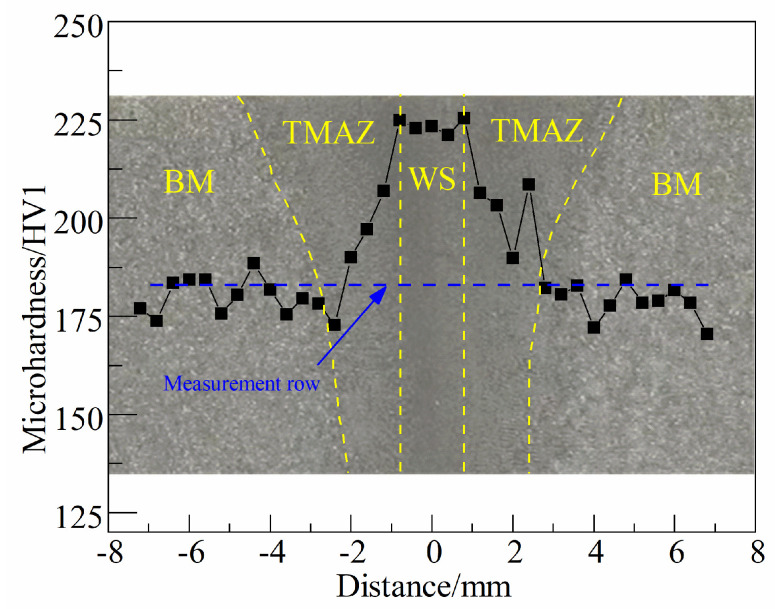
Microhardness distribution of S355 inertia friction-welded joint.

A transverse microhardness distribution test was carried out 5 mm below the weld’s outer surface, with the blue dotted line in Figure 9 showing the exact position of the measurement row.

Figure 9 clearly depicts the variation pattern of microhardness from WS to BM, and the WS area has the highest microhardness due to the thermal processing mechanism of the inertia friction welding process, consequently promoting the formation of granular bainite, reaching an average of 233 HV. The thermal coupling effect of inertia friction welding is comparatively reduced in the TMAZ area compared to the WS area, which partly includes granular bainite and F+P, so microhardness in the TMAZ area gradually decreases as the test position deviates from the center, while hardness in the BM remains more stable. Finally, the BM microhardness is lowest because there is no supercooled structure in this area.

#### 3.2.2. Tensile Strength

**Figure 10:**
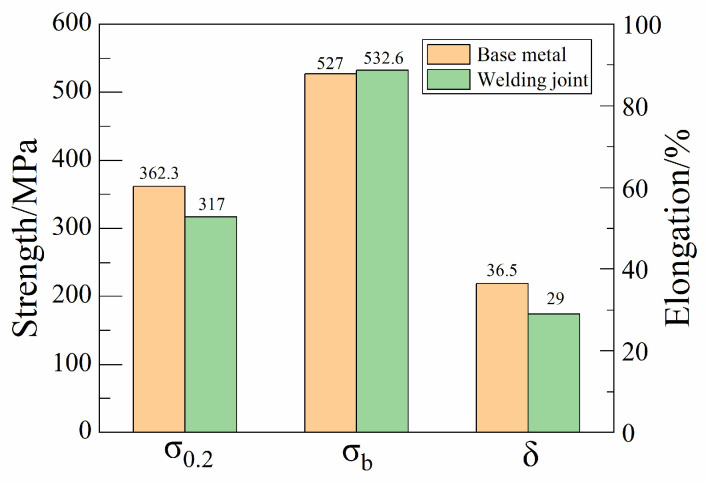
Tensile properties of base metal and welded joint.

As shown by the test results in Figure 10, the yield strength of the welded joint achieved 87.5% of the base material level, which was 317 MPa. The tensile strength of the welded joint was similar to that of the base material. Additionally, the elongation reached 79.5% of the base material level.

**Figure 11:**
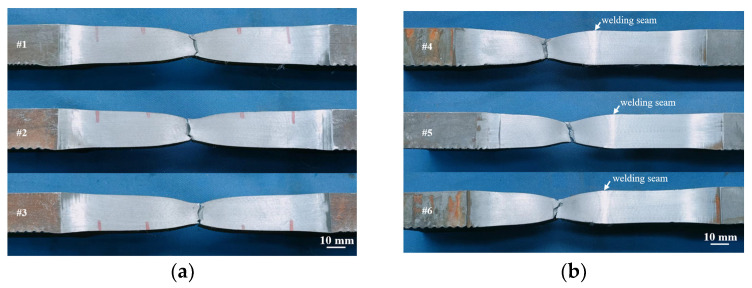
Lateral macroscopic morphology of full-thickness tensile specimens: (**a**) base metal; (**b**) welded joint.

In Figure 11 we can see that the tensile specimens of the parent material fractured at the midpoint, whereas the tensile specimens of the welded joint fractured in the BM zone away from the center, indicating that the inertia friction weld was subjected to a strengthening mechanism and its strength exceeded that of the parent material. The differential microstructure distribution of the joints did not lead to tensile fracture in the vicinity of the weld.

#### 3.2.3. Bend Test

**Figure 12:**
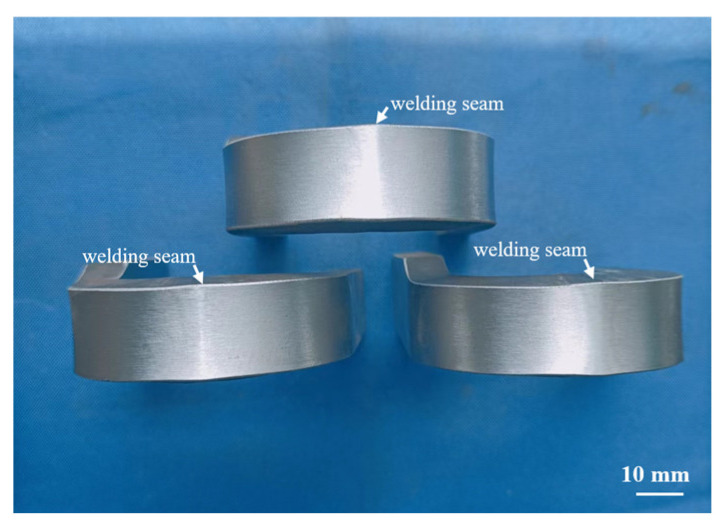
Side bending specimens of S355 inertia friction-welded joint.

To investigate the ductility of inertia friction-welded joint, a three-point side bend test was carried out on specimens 10 mm thick with a 40 mm diameter bending core, at a bending angle of 180°.The bending test results are illustrated in Figure 12, demonstrating exceptional ductility of the welded joints as there are no notches or cracks on either the entire tensile surface or the edges.

#### 3.2.4. Impact Test

The Charpy (V-notch) pendulum impact test method was utilized to ascertain the energy absorption of both the base metal and welded joints. Experiments were conducted at both room temperature and in a −40 °C environment, utilizing samples measuring 10 mm × 10 mm × 55 mm, and at the sampling positions of the BM, TMAZ and WS, respectively.

**Figure 13:**
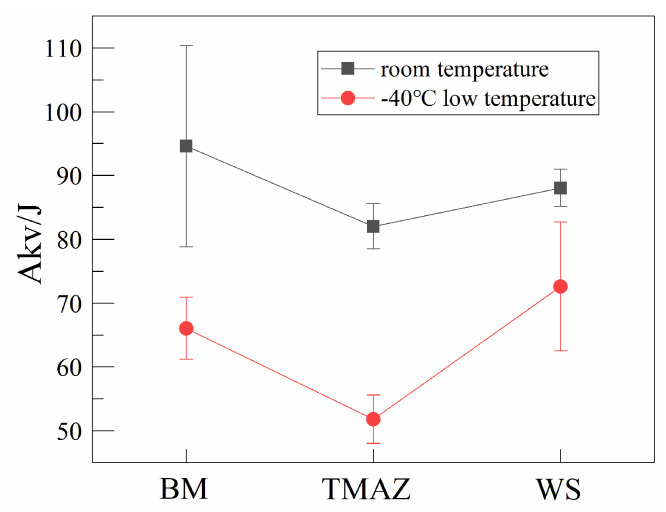
Room-temperature and −40 °C low-temperature impact test results.

The test results are shown in Figure 13. From the results, it can be seen that in a −40 °C environment, the impact toughness decreased significantly in different zones. The impact toughness of both the BM and TMAZ decreased by an average of 30 J at −40 °C, whereas the impact toughness of the WS was only reduced by 15 J due to the −40 °C low-temperature effect. It was also found that the impact toughness of TMAZ was always lower than that of BM and WS, both at room temperature and at −40 °C. Remarkably, the impact toughness of WS was higher than that of BM at −40 °C.

## 4. Discussion

Based on the test results presented above, it is observed that the S355 inertia friction-welded joints are free of any weld defects and display desirable tensile and ductile properties similar to the base material. The microhardness results indicate that the weld wasstrengthened by hot working.

Under the effect of the thermo-mechanical coupling of IFW, the microstructure of WS and TMAZ produced significant changes compared to BM, exhibiting both supercooled austenite phase transition characteristics and recrystallisation characteristics after plastic deformation. During the welding process, WS heats up to a red-hot state [26,27], leading to complete austenitization. Due to the cold source of the base material, a significant degree of subcooling occurs, leading to the formation of a microstructure consisting mainly of granular bainite, alongside some acicular ferrite and pearlite. The TMAZ region undergoes both austenitic transformation and recrystallization processes. The former results in a granular bainite structure, while the latter leads to the formation of F+P equiaxed fine grains.

The welded joint contains bainite, resulting in strength and hardness superior to those of the base material, with a tensile strength of 532.6 MPa. However, the elongation of and reduction in the area are lower than those of the base material, at 87.5% and 79.5%, respectively. Nevertheless, the welded joint exhibits sufficient ductility reserve, as evidenced by the lack of cracking during transverse side bending.

In contrast to the tensile and ductile properties, the distribution pattern of room-temperature and −40 °C impact toughness of the IFW joints is more complex. The impact toughness of WS at room temperature is slightly lower than that of BM, but the −40 °C impact toughness exceeds that of BM. Since the granular bainite in the WS zone is a uniformly distributed multi-phase microstructure, plus the acicular ferrite exhibits a random orientation, the low-temperature toughness is increased. But unlike WS, TMAZ exhibits a relatively poor level of impact toughness, both at room temperature and at −40 °C. This is due to the fact that TMAZ exhibits an inhomogeneous band feature, and the impact toughness is affected by the directionality of the bands.

Inertia friction welding can be used to easily weld thick-walled tubular steel structures without defects in a single pass, provided the welding equipment has sufficient forging force, which always requires high financial inputs. The thermal processing improvement mechanism can ensure that the inertia friction welding head has excellent tensile and good ductile properties, so the method has broad development potential in the manufacture of thick-walled rotating-body workpieces. It is necessary to verify the fatigue performance of welded joints and to evaluate post-weld residual stresses in an effort to provide more complete performance data for engineering applications.

## 5. Conclusions

(1) The inertia friction welding method was used to achieve the one-step formation of a bogie crossmember ring joint with a diameter of 169 mm and a thickness of 20.5 mm, and the macroscopic metallurgical results show that there are no visible defects within the weld.
(2) The WS undergoes a complete austenitization process, and the post-weld microstructure characteristics are mainly influenced by the degree of subcooling brought about by the cold source of the base metal, resulting in the formation of granular bainite and acicular ferrite, plus a small amount of pearlite.
(3) The TMAZ microstructure is affected by the dual mechanism of austenitic phase transformation and recrystallization after plastic deformation, finally forming granular bainite bands and F+P bands.
(4) Due to the presence of bainite in the welded joint, the strength and hardness of the joint exceed those of the base material, and the tensile strength reaches 532.6 MPa. But the elongation of and reduction in the area reduced compared with the base material, respectively, reach 87.5% and 79.5% of the base metal. Even so, the transverse side bending of the welded joints do not crack, indicating that the welded joint has sufficient ductility reserve.
(5) The room-temperature impact toughness of WS is slightly lower than that of BM, but the −40 °C impact toughness exceeds that of BM. Compared to WS, the impact toughness of TMAZ is significantly lower than that of the parent material at both room and −40 °C low temperatures.

## Figures and Tables

**Table 1:** Chemical composition and mechanical properties of S355 steel.

Element	C	Mn	Si	Cr	Ni	P	S	Fe	Rp0.2 /MPa	Rm /MPa
wt%	0.05	1.60	0.25	0.04	0.03	0.018	0.004	Ba	≥300	≥490

## Data Availability

Data is contained within the article.

## References

[1] Ilesanmi D., Khumbulani M., Festus F., Adefemi A. (2020). Numerical simulation and experimental validation of the welding operation of the railcar bogie frame to prevent distortion. Int. J. Adv. Manuf. Technol..

[2] Lu Y.H., Lu C., Zhang D., Chen T., Zeng J., Wu P.B. (2019). Welding simulation of railway bogie frame side beam: Analyses of residual stresses, clamping forces, distortion and prediction of fatigue S-N curves. J. Manuf. Process..

[3] Shukri A., Kaylan M., Kamen U. (2023). Numerical computation methods of welding deformation and their application in bogie frame for high-speed trains. Proc. Inst. Mech. Eng. Part F J. Rail Rapid Transit.

[4] Gao Y.H., Zhao W.Z. (2014). Adaptive optimization with weld fatigue constraints based on surrogate model for railway vehicles. Mech. Based Des. Struct. Mach..

[5] Seo J.W., Kwon S.J., Lee C.W., Lee D.H., Goo B.C. (2021). Fatigue strength and residual stress evaluation of repair welding of bogie frame for railway vehicles. Eng. Fail. Anal..

[6] Zhao X.Y., Xie S.Q., Zhang Y.L., Li Q., Wang W.J., Wang B.J. (2022). Fatigue reliability analysis of metro bogie frame based on effective notch stress method. Eng. Fail. Anal..

[7] Yang G.X., Wang M., Li Q., Li Q., Ding R. (2019). Methodology to Evaluate Fatigue Damage of High-Speed Train Welded Bogie Frames Based on On-Track Dynamic Stress Test Data. Chin. J. Mech. Eng..

[8] Mao L.Y., Wang W.J., Liu Z.M., Sha M., Zhang D.Y. (2022). Investigation of the fatigue crack growth behavior of S355 steel weldments of motor hangers of high-speed trains. Eng. Fail. Anal..

[9] Dariusz R., Janusz L., Grzegorz L., Zbigniew M., Jose A.C., Wojciech M. (2022). The energy approach to fatigue crack growth of S355 steel welded specimens subjected to bending. Theor. Appl. Fract. Mech..

[10] Hassan A.K. (2022). Material and residual stress improvement in S355 welded structural steel using mechanical and thermal post-weld treatment methods. Steel Constr..

[11] Boris F., Ivan L., Davor S., Mateo G. (2022). Fatigue tests of as-welded and HFMI treated S355 details with longitudinal and transverse attachments. Weld. World.

[12] Gu B.P., Wang P., Hu X., Jin Z.D., Lai J.T., Xu G.H., Yang Z.S., Huo Z.P., Wang Z.S. (2021). Effects of High Frequency Impact Vibration Stress Relief on Residual Stress and Microstructure of a Laser Surface Treated S355 Steel. Lasers Eng..

[13] Wu L.J., Chen J., Xu L., Wu X.Y., Xia C.Y., Wu C.S., Wang Z.S. (2023). Study on GMAW assisted by compound external magnetic fields in bogie manufacturing with T-joints and single-bevel grooves. Weld. World.

[14] Wang F.X., Yang X.Q., Yin Y.Y., Cui L. (2017). Thermal process influence on microstructure and mechanical behavior for friction taper plug welding in structural steel S355. Int. J. Adv. Manuf. Technol..

[15] Zhang X.D., Deng C.Y., Wang D.P., Wang Z.J., Teng J.H., Cao J., Xu W., Yang F. (2016). Improving bonding quality of underwater friction stitch welds by selecting appropriate plug material and welding parameters and optimizing joint design. Mater. Des..

[16] Cui L., Yang X.Q., Wang D.P., Hou X.P., Cao J., Xu W. (2014). Friction taper plug welding for S355 steel in underwater wet conditions: Welding performance, microstructures and mechanical properties. Mater. Sci. Eng. A.

[17] (2019). Metallic Materials—Tensile Testing—Part 1: Method of Test at Room Temperature.

[18] (2023). Destructive Tests on Welds in Metallic Materials—Bend Tests.

[19] (2022). Destructive Tests on Welds in Metallic Materials—Impact Tests—Test Specimen Location, Notch Orientation and Examination.

[20] Sun J.M., Hensel J., Klassen J., Nitschke-Pagel T., Dilger K. (2019). Solid-state phase transformation and strain hardening on the residual stresses in S355 steel weldments. J. Mater. Process. Technol..

[21] Rodrigues D.M., Leitao C., Balakrishnan M., Craveiro H.D., Santiago A. (2021). Tensile properties of S355 butt welds after exposure to high temperatures. Constr. Build. Mater..

[22] Balakrishnan M., Leitao C., Craveiro D., Rodrigues D.M., Santiago A., Silva L.S., Subramanian C. (2022). Post fire tensile properties of S355 J2 structural steel welded connections for construction industrial applications. Metall. Res. Technol..

[23] Stornelli G., Gaggiotti M., Gattia D.M., Schmidt R., Sgambetterra M., Tselikova A., Zucca G., Di Schino A. (2022). Vanadium Alloying in S355 Structural Steel: Effect on Residual Austenite Formation in Welded Joints Heat Affected Zone. Acta Metall..

[24] Amborish B., Michail N., Laurie Da S., Ryan O., Salaheddin R. (2022). Continuous Drive Friction Welding of AISI 8630 Low-Alloy Steel: Experimental Investigations on Microstructure Evolution and Mechanical Properties. J. Manuf. Sci. Eng. Trans. ASME.

[25] Selvaraj R., Shanmugam K., Selvaraj P., Prasanna N.B., Balasubramanian V. (2023). Optimization of mechanical properties of rotary friction welding parameters of low alloy steel tubes using design of experiments concept. Int. J. Interact. Des. Manuf..

[26] Zhang W.C., Deng Y.F., Zhan P.F., Zeng J.C., Xiao X.P. (2022). Effects of joint heat distribution on material flow and microstructure in continuous drive friction welding of 45 # steel. Proc. Inst. Mech. Eng. Part C J. Mech. Eng. Sci..

[27] Kim Y., Kim D., Park J., Song K. (2022). Implementation of Exceptional Microstructures and Mechanical Properties of Structural Carbon Steel Tubes by Friction Welding. Mater. Trans..

